# RANKL-dependent osteoclast differentiation and gene expression in bone marrow-derived cells from adult mice is sexually dimorphic

**DOI:** 10.1016/j.bonr.2023.101697

**Published:** 2023-07-01

**Authors:** Suchita Desai, Pernilla Lång, Tuomas Näreoja, Sara H. Windahl, Göran Andersson

**Affiliations:** a Karolinska Institutet, Department of Laboratory Medicine - Division of Pathology, Huddinge, Sweden; b Department of Life Technologies, University of Turku, Finland

**Keywords:** Bone marrow cells, Adhesion, Differentiation, Gene expression, Sex, Osteoclasts

## Abstract

Sex-specific differences in bone integrity and properties are associated with age as well as the number and activity of cells involved in bone remodeling. The aim of this study was to investigate sex-specific differences in adhesion, proliferation, and differentiation of mouse bone marrow derived cells into osteoclasts. The adherent fraction of bone marrow- derived cells from 12-week-old male and female C57BL/6J mice were assessed for their adhesion, proliferation, and receptor activator of nuclear factor κB (RANKL)-induced differentiation into osteoclasts. Female bone marrow derived macrophages (BMDMs) displayed higher adhesion and proliferation ratio upon macrophage colony stimulating factor (M-CSF) (day 0) and M-CSF + RANKL (day 4) treatment, respectively. On the contrary, male BMDMs differentiated more efficiently into osteoclasts upon RANKL-treatment compared to females (day 5). To further understand these sex-specific differences at the gene expression level, BMDMs treated with M-CSF (day 0) and M-CSF + RANKL (day 4), were assessed for their differential expression of genes through RNA sequencing. M-CSF treatment resulted in 1106 differentially expressed genes, while RANKL-treatment gave 473 differentially expressed genes. Integrin, adhesion, and proliferation-associated genes were elevated in the M-CSF-treated female BMDMs. RANKL-treatment further enhanced the expression of the proliferation- associated genes, and of genes associated with inhibition of osteoclast differentiation in the females, while RANK-signaling-associated genes were upregulated in males. In conclusion, BMDM adhesion, proliferation and differentiation into osteoclasts are sex-specific and may be directed by the PI3K-Akt signaling pathway for proliferation, and the colony stimulating factor 1-receptor and the RANKLsignaling pathway for the differentiation.

## Highlights

• Source of precursors contributes to sexual dimorphism in terms of adhesion and differentiation into osteoclasts
• Female MCSF-treated bone marrow- derived cells adhere more and show continued proliferation upon RANKL treatment
• Male bone marrow- derived cells differentiate more efficiently into osteoclasts than female cells
• PI3K-Akt and CSF1R-RANKL pathways are important for sex differences in adhesion/proliferation, and differentiation.

## 1 Introduction

A healthy bone has balanced bone remodeling i.e. balanced activities of bone resorbing osteoclasts and bone forming osteoblasts (Sims and Gooi, 2008). Any imbalance in this state can lead to various bone-associated disorders such as osteoporosis or osteopetrosis, and the development of these disorders, as well as normal bone development, varies between the sexes and with age. Several studies have shown differences in site-specific bone mineral density between the sexes (Warming et al., 2002; Nieves et al., 2005; Hannan et al., 2000). Premenopausal women experience a decrease in site-specific bone mineral density that decreases further after menopause. In men, the site-specific bone loss is minimal and takes place throughout their life. This age-related bone loss is accentuated after 50 years of age (Warming et al., 2002). These sex- specific differences in bone are a result of genetics, hormones, age, and the environment that together affect the osteoclast (OC) number and their resorptive activity.

Osteoclasts are multinucleated cells derived from the hematopoietic stem cell (HSC) lineage via monocytes and macrophages (Sims and Gooi, 2008). The classical signaling for OC differentiation from its precursors is mediated via the binding of the macrophage colony stimulating factor (M-CSF) and receptor activator of nuclear factor κB ligand (RANKL) and to their receptors, colony stimulating factor 1-receptor (CSF1R) and receptor activator of nuclear factor κB (RANK) respectively. This activates the NFκB signaling pathway and other downstream mediators and promotes OC differentiation (Teitelbaum, 2000).

In vitro studies for osteoclastogenesis have given an insight into the process of differentiation and show either high or low osteoclast generation between the sexes, with age as well as culture conditions. Some studies have shown that female OC-precursor cells differentiate more avidly into osteoclasts (Mun et al., 2021; Paglia et al., 2016; Abe and Aoki, 1989), while others have shown that male cells differentiate more efficiently (Valerio et al., 2017). Valerio and colleagues have shown that upon LPS stimulation, different populations of osteoclast progenitors from males differentiated more readily into osteoclasts than the same population from females (Valerio et al., 2017).Another study showed that male bone marrow-derived macrophages are more migratory in response to leptin and depict a more pro-inflammatory environment (Chen et al., 2021). On the contrary, females displayed higher osteoclast number compared to the males upon the loss of *Runx1* (Paglia et al., 2016), and the activation of a specific osteoclast precursor in females is enhanced due to an inflammatory environment (Mun et al., 2021). Besides, another study has indicated that the sex hormones in males could result in lower OC number and resorbed area in the femur, compared to female mice (7 and 14 week old) (Abe and Aoki, 1989). These discrepancies may be due to the source of the osteoclast precursors, age, species and strain of the mice or the culturing techniques used, which in turn could affect the genes expressed by the osteoclast precursors and regulation of their pathways.

For this purpose, we investigated, as a source of precursors, bone marrow cells that have not been selected for any specific markers. An insight into the sex- and RANKL treatment-specific differences in these cells could contribute to the existing knowledge on osteoclasts and their activity, that if disturbed could eventually lead to sex-specific disorders. Hence the aim of this study was to investigate sex- and RANKL treatment-specific differences between adhesion, proliferation, and differentiation of bone marrow derived macrophages (BMDM) into osteoclasts.

## 2 Material and methods

### 2.1 Animals

12-week-old male and female C57BL/6J wildtype mice were obtained by breeding heterozygous TRAP colony, a kind gift from from the Egesten lab, Lund University, and were housed in a standard animal facility at the KMB, Biomedicum, Karolinska Institutet, Sweden under controlled temperature (22 °C) and photoperiod (12 h of light, 12 h of dark) and fed pellet diet (CRM(P) and Special diet service UK) ad libitum. The animals were mixed in the cages and no control group was assigned as the aim was to compare the sexual dimorphism. The study was approved by the local Ethical Committees for Animal Research (Ethical number 8387-2018) and complied with national and ARRIVE guidelines, and the EU Directive 2010/63/EU for animal experiments.

### 2.2 Bone marrow extraction and culture

Bone marrow from 12-week-old male (*N* = 7) and female (*N* = 10) wild type mice was collected from the femur cavity by flushing with complete alpha MEM media-with phenol red (Gibco) containing 10 % fetal bovine serum-not charcoal stripped (FBS) (Thermo Fischer scientific), 1 % pen strep (Gibco) and 1 % glutamax (Gibco), with a syringe. After centrifugation, the cells were resuspended in complete alpha-MEM and cultured in the presence of 25 ng/ml of mouse macrophage colony stimulating factor (M-CSF) (R&D systems). On the next day, the media and non-adherent cells were discarded, and the adherent cells were cultured in the presence of media supplemented with MCSF until confluency (5–7 days). Upon confluency the BMDM were collected with accutase (Merck) treatment and frozen down in 5 % dimethyl sulfoxide (DMSO) (Sigma Aldrich) and fetal bovine serum (FBS) until the experiment.

### 2.3 Cyquant assay for adhesion and proliferation

For the adhesion assay, male and female BMDMs were thawed and cultured at a concentration of 30,000 cells/cm^2^ in the presence of alpha MEM complete medium with 25 ng/ml of MCSF for 5 h. For the proliferation assay, male and female BMDMs were cultured for 4 days at a concentration of 30,000 cells/cm^2^ in the presence of alpha MEM complete medium with a combination of 25 ng/ml of MCSF and 10 ng/ml of RANKL (Peprotech). At the end of the culture period for adhesion (5 h) and proliferation (4 days), the cells were analyzed using the CyQUANT™ NF Cell Proliferation Assay (Thermo Scientific) according to the manufacturer's protocol. Fluorescence intensity was measured as a 15 ∗ 15 well matrix scan using the CLARIOstar spectrophotometer (BMG LABTECH).

### 2.4 Osteoclast differentiation

To study osteoclast differentiation, male and female BMDMs were cultured at a concentration of 30,000 cells/cm^2^ for 2, 3, 4, 5, and 6 days in the presence of alpha MEM complete medium supplemented with a combination of 25 ng/ml of MCSF and 10 ng/ml RANKL (referred as MRL or RANKL treatment). At the end of the respective culture days, the cells were fixed with 4 % PFA and stored in PBS until cathepsin K staining. For cathepsin K staining, antigen retrieval was performed by heating the samples at approximately 95–100°C with citrate buffer for 15–30 s. This was followed by blocking with H_2_O_2_ for 5 min and blocking in goat serum (1:5 PBST) (DAKO) for 30 min. The wells were incubated with cathepsin K antibody (diluted 1:700 in 1%BSA in PBST) (Agrisera) at 4 °C, overnight. This was followed by incubation with biotinylated goat anti-rabbit antibody (diluted 1:800 in PBS) (DAKO) for 2 h at room temperature. Detection was performed using the VectaStain ABC kit followed by DAB staining. Cathepsin K-stained cells were imaged using NIKON microscope (BergmanLabora) equipped with a digital camera. The number of osteoclasts, defined as Cathepsin stained cells with three or more nuclei, were counted using the Osteomeasure (OsteoMetrics) software. The investigator was blinded to the identity of the samples during counting.

### 2.5 RNA extraction

Based on adhesion, proliferation, and differentiation data, we wanted to study the differential gene expression. Female and male BMDMs were cultured at a concentration of 30,000 cells/cm^2^ in the presence of alpha MEM complete medium supplemented with 25 ng/ml of MCSF (for 12 h) or a combination of 25 ng/ml of MCSF and 10 ng/ml of RANKL (for 4 days). The RNA lysates were collected for sequencing purpose either on day 0 or 4 in beta mercaptoethanol: RLT plus buffer (1:100) (QIAGEN) and stored at −20°C until RNA extraction. Total RNA extraction was performed using RNAeasy Mini kit (QIAGEN) according to the manufacturer's instructions, and the RNA concentration was quantified using Nanodrop (Thermo Scientific) and stored at −80 °C until sequencing.

### 2.6 RNA sequencing

Of the extracted RNA samples, five mice per sex and treatment were considered eligible for mRNA sequencing (minimum concentration of 20 ng/ml) which was performed by Novogene (UK). The groups for sequencing were as follows: day 0 MCSF treated males (control male/CM), day 0 MCSF treated females (control female/CF), day 4 RANKL treated males (treated male/TM), and day 4 RANKL treated females (treated female/TF). Based on these groups the comparison was done between CM vs CF, TM vs TF, TM vs CM and TF vs CF groups. After library preparation and quality control, the RNA was sequenced using the Illumina platform. The clean reads were mapped to the reference genome (*mus musculus*) using Hisat2 v2.0.5. Gene expression level quantification was done using featureCounts v1.5.0-p3 to count the read numbers mapped to each gene. Differential expression analysis was performed for biological replicates using the DESeq2 R package (1.20.0). Genes with an adjusted *p*-value ≤0.05 and log2fold change >2 were assigned as differentially expressed. Enrichment analysis of differentially expressed genes was studied using Kyoto Encyclopedia of Genes and Genomes (KEGG) database. ClusterProfiler R package was used to test the statistical enrichment of differential expression genes in KEGG pathways. The DESeq2 output of normalised gene counts from TM vs CM and TF vs CF comparison were used for plotting the data and performing statistical analysis for studying expression of genes specific to our study.

### 2.7 Statistical analysis

Statistical analysis was performed using the GraphPad Prism version 9. Normal distribution of data was assessed. Adhesion, proliferation, and osteoclast differentiation were assessed using the non-parametric Mann Whitney test. For adhesion and proliferation analysis an N of four and five were used for female and male, respectively. This was done to avoid the inter-plate variation while adjusting to females and exclude the animals that were cultured on different plates. Two-way ANOVA assuming equal variability of differences, followed by Tukey's multiple comparison was performed on the normalised gene count output from RNA sequencing for studying the difference between the sexes with the treatments. A *p*-value of ≤0.05 was considered significant.

## 3 Results

### 3.1 Female MCSF-treated bone marrow-derived cells adhere more and show continued proliferation upon RANKL treatment

**Fig. 1:**
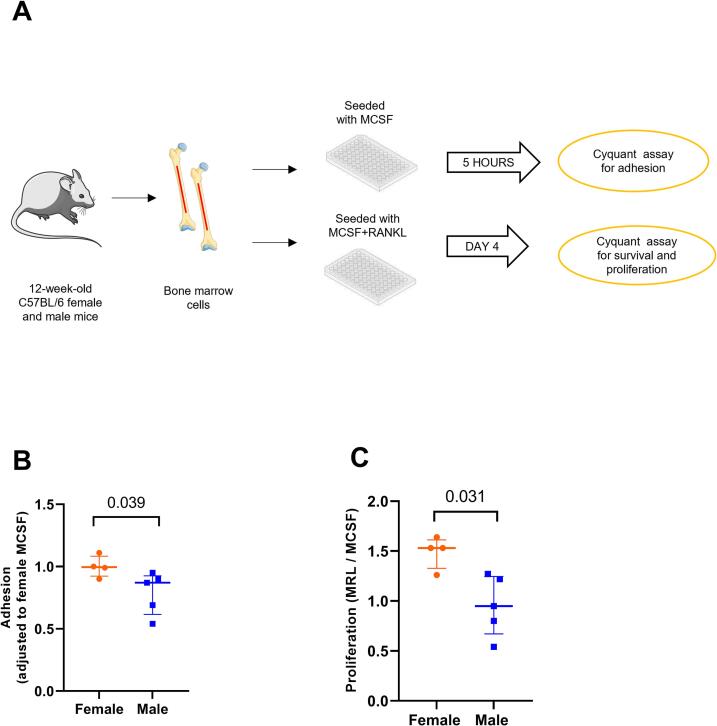
Bone marrow derived macrophages from females adhere and proliferate more than male macrophages as detected by the Cquant cell proliferation assay. (A) Schematic representation showing bone marrow-derived cell culture for adhesion and proliferation assay. (B) Adhesion of female and male bone marrow cells treated with M-CSF, measured 5 h after seeding. All data is adjusted to female (M-CSF) fluorescence output. (C) Proliferation of female and male bone marrow cells shown as a ratio of MRL/M-CSF treatment measured on day 4. All data is adjusted to female (M-CSF) fluorescence output. (B, C) Non-parametric Mann Whitney's test was performed. Results are shown as median, min, max, to show all points (B, C), *N* = 4–5. (A) The figure was partly generated using Servier Medical Art, provided by Servier, licensed under a Creative Commons Attribution 3.0 unported license.

Cells isolated from female and male mouse bone marrow were cultured with M-CSF and MRL to check for adhesion and proliferation using the CyQUANT™ NF Cell Proliferation Assay. The number of adherent cells in female and male M-CSF-treated bone marrow cells was measured. (Fig. 1A). BMDMs from female mice adhered more when compared to the male BMDMs (+21 %, Fig. 1B). To investigate the proliferative capacity of these adhered cells upon RANKL treatment, M-CSF- and MRL-treated cells were cultured until day 4 and the MRL/M-CSF ratio was assessed (Fig. 1A). No difference in proliferation was seen between the M-CSF or MRL treatments when comparing the two sexes (Fig. S1A). However, female cells exhibited a higher MRL/M-CSF-ratio (+53 %) than male cells (Fig. 1C). This higher ratio in female cells on day 4 is due to a non-significantly lower proliferation in the M-CSF-treated group (Fig. S1A).

### 3.2 Male bone marrow-derived cells differentiate more efficiently into osteoclasts than female cells

**Fig. 2:**
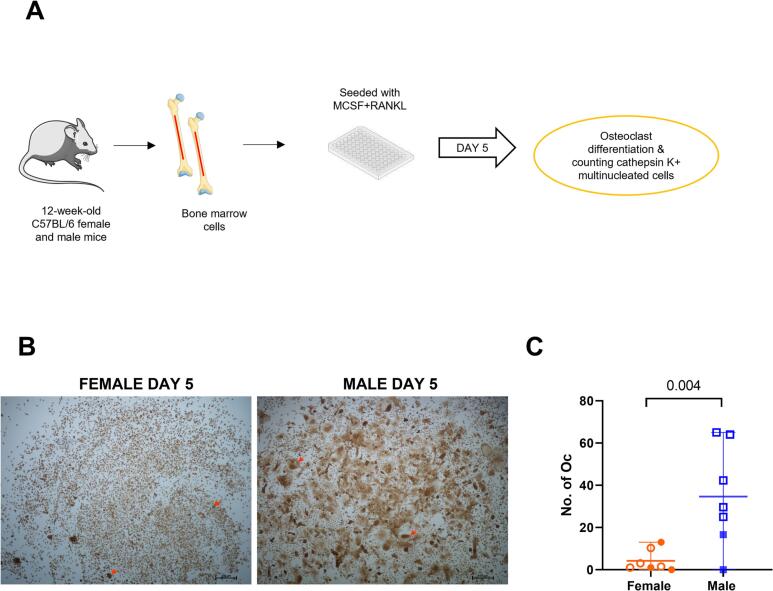
Male bone marrow-derived macrophages differentiate faster into osteoclasts than female macrophages (A) Schematic representation showing bone marrow-derived cell culture for osteoclast differentiation. (B) Cathepsin K-stained male and female osteoclasts on day 5. Red arrows indicate representation of cells counted as osteoclasts. (C) Number of osteoclasts (three or more nuclei) counted manually on day 5. Non-parametric Mann Whitney's test was performed. Results are shown as median, min, max, to show all points *N* = 7–10. Empty circles and squares indicate the samples that were sent for mRNA sequencing. (A) The figure was partly generated using Servier Medical Art, provided by Servier, licensed under a Creative Commons Attribution 3.0 unported license.

We then investigated if there was a sex-specific difference in differentiation into osteoclasts. MRL-stimulated cathepsin K-positive multinucleated cells were counted on day 5 (Fig. 2A). Interestingly, male cells differentiated more efficiently into osteoclasts as evident by an 8-fold increase in the number of cathepsin K-stained, multinucleated cells (three or more nuclei) when compared to female cells (Figs. 2B, C and S1B).

### 3.3 mRNA gene expression using DESeq2

**Fig. 3:**
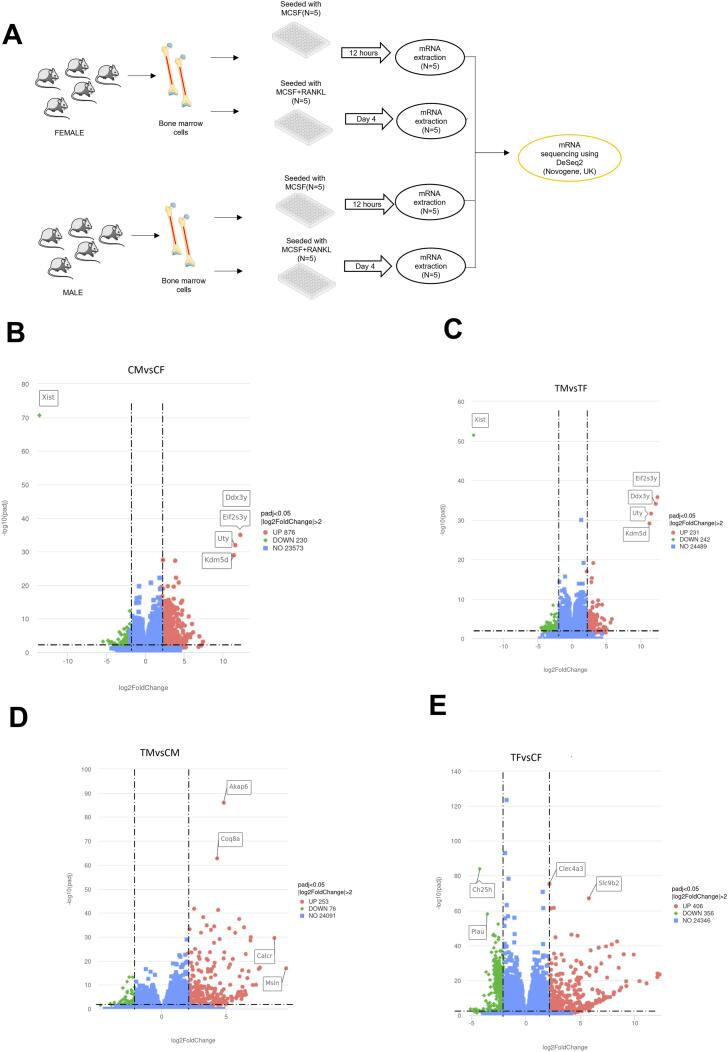
Differential expression of genes was seen upon bulk mRNA sequencing. (A) Schematic representation showing bone marrow derived cell culture for mRNA sequencing using DESeq2 R package (1.20.0). Volcano plot shows differentially expressed genes for (B) control male (CM) vs control female (CF), (C) treatment male (TM) vs treatment female (TF), (D) treatment male (TM) vs control male (CM), and (E) treatment female (TF) vs control female (CF). Control group indicates M-CSF stimulation only, while treatment group indicates M-CSF + RANKL stimulation for 4 days. The dotted lines indicate the threshold for log2fold change (x-axis) and p-adjusted value (y-axis) of >2 and <0.05, respectively. *N* = 5.UP indicates upregulation, DOWN indicates down regulation, NO indicates no difference as per the threshold values. (A) The figure was partly generated using Servier Medical Art, provided by Servier, licensed under a Creative Commons Attribution 3.0 unported license.

Since females adhered more on day 0, while the males differentiated more on day 4, it was of interest to assess possible differences between the sexes at the gene expression level. For this purpose, mRNA sequencing was performed on control (pre-osteoclasts = 12 h of M-CSF treatment) and treated (committed OC differentiating cells = 4 days of MRL treatment) cells from female and male mice (Fig. 3A).

DESeq2 based differential expression analysis (threshold of log2 fold >2 and padj <0.05) gave 1106 (876 up, 230 down) differentially expressed genes for control male (CM) vs female (CF) (Figs. 3B, S2), 473 (231 up, 242 down) genes for treatment male (TM) vs female (TF) (Figs. 3C, S2), 329 (253 up, 76 down) genes for treatment male (TM) vs control male (CM) (Figs. 3D, S2), and 762 (406 up, 356 down) genes for treatment female (TF) vs control female (Figs. 3E, S2). As expected, the X-linked gene, Xist, was strongly reduced and Y-linked genes (Eif2s3y, Ddx3y, Uty and Kdm5d) were elevated in male compared to female cells (Figs. 3B and C).

### 3.4 Enrichment pathway analysis

Upon KEGG enrichment based on the upregulated genes, only the CM vs CF group had significantly enriched pathways based on the 876 upregulated genes shown by its volcano plot (Figs. 3A, S3A), while the other comparisons did not have enough power to detect significant differences due to the fewer upregulated genes (Fig. S3B-D).

Of the significantly enriched pathways in the CM vs CF group, extracellular matrix (ECM)-receptor interaction, calcium signaling pathway, PI3K-Akt signaling pathway, focal adhesion and cytokine-cytokine receptor interaction were of interest to our study as they could help understanding the sexual dimorphism seen in cell adherence which further contributes to the difference seen in differentiation (Table S1). Of these pathways the most recurring genes (highlighted in red in Table S1) were integrins and collagen family members.

Further, to assess the difference in gene regulation upon RANKL treatment between the sexes, KEGG enrichment analysis between the TM vs TF group was done based on the 231 upregulated genes. No pathways were significantly altered in this comparison based on the threshold (Fig. S4, Table S2).

Since osteoclast differentiation differed between the sexes and was also one of the hits in both the TM vs CM and the TF vs CF comparisons (Fig. S3C and D), however not significant, the genes involved in its KEGG pathway were studied (Table S3). Functional genes like acid phosphatase 5, tartrate resistant (Acp5), cathepsin K (Ctsk), integrin subunit beta 3 (Itgb3), osteoclast associated Ig-Like receptor (OSCAR) and calcitonin receptor (Calcr) were upregulated in both sexes. Paired-Ig-like receptor A2 (Pira2) (negative regulation of osteoclast differentiation), (FOS Like 2, AP-1 Transcription Factor Subunit) Fosl2 and *FosB* proto-oncogene, AP-1 transcription factor subunit (FosB) (positive regulation of osteoclast differentiation) were regulated in the females only, while NADPH oxidase 1 (Nox1) (positive regulation of osteoclast differentiation) and TNF superfamily member 11 (Tnfsf11) (regulation of osteoclast differentiation) were regulated only in the males (Table S3).

**Fig. 4:**
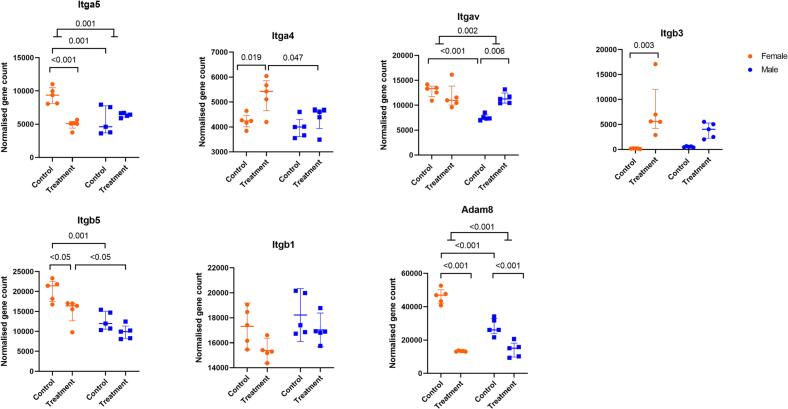
DESeq2 output of normalised gene counts shown for genes involved in adhesion Control group indicates M-CSF stimulation only, while treatment group indicates M-CSF + RANKL stimulation for 4 days. Two-way ANOVA assuming equal variability of differences followed by Tukey's multiple comparison test was performed and only significant values are shown on the graphs. The interaction effect indicates that the change in the control versus treatment group for each sex is significantly different between the sexes. Results are shown as median with interquartile range to show all points, N = 5.

**Fig. 5:**
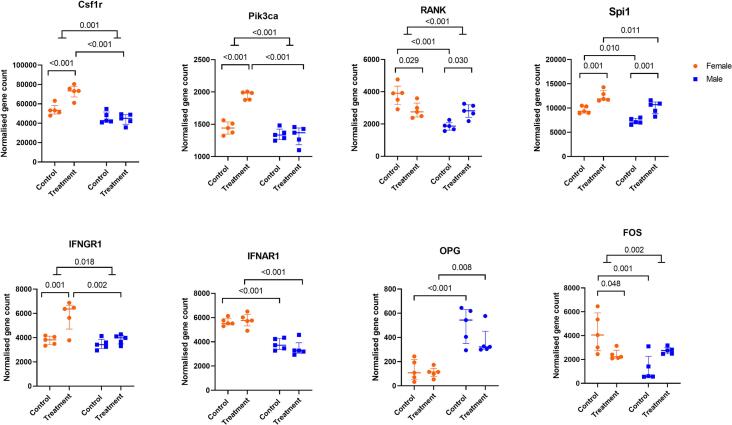
DESeq2 output of normalised gene count is shown for genes associated with proliferation and early differentiation genes. Control groups indicates M-CSF stimulation only, while treatment groups indicate M-CSF + RANKL stimulation for 4 days. For each group a two-way ANOVA assuming equal variability of differences followed by Tukey's multiple comparison test was performed and only significant values are shown on the graph. The interaction effect indicates that the change in the control versus treatment group for each sex is significantly different between the sexes. Results are shown as median with interquartile range to show all points, N = 5.

**Fig. 6:**
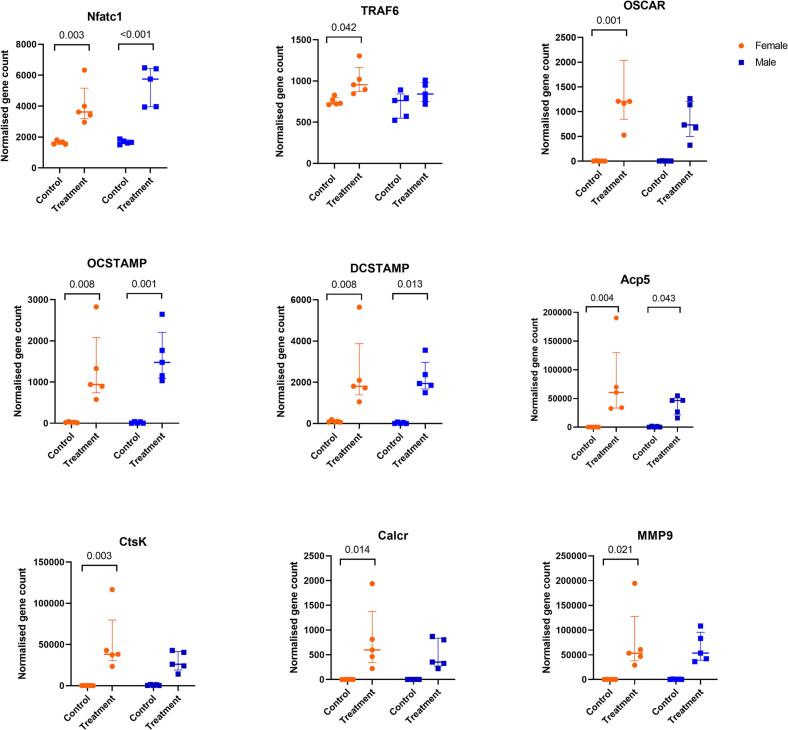
DESeq2 output of normalised gene counts shown for late and function genes in osteoclast differentiation. Control group indicates M-CSF stimulation only, while treatment group indicates M-CSF + RANKL stimulation for 4 days. For each group, a two-way ANOVA assuming equal variability of differences followed by Tukey's multiple comparison test was performed and only significant values are shown on the graph. The interaction effect indicates that the change in the control versus treatment group for each sex is significantly different between the sexes. Results are shown as median with interquartile range to show all points, N = 5.

With this information from the KEGG analysis, we further wanted to assess in detail the sex- and RANKL treatment-specific differences in the transcript levels of selected adhesion and differentiation-associated genes in the CM, CF, TM, and TF groups. Based on existing literature and KEGG analysis, we selected integrin and adhesion genes as well as genes associated with osteoclast differentiation (Fig. 4, Fig. 5, Fig. 6).

### 3.5 Adhesion-associated genes

First, sex-specific gene expression differences of adhesion genes between pre-osteoclast cells (M-CSF treated cells) and OC differentiating cells (MRL-treated cells) were examined. Integrin Subunit Alpha 5 (Itga5), Integrin Subunit Alpha V (Itgav), Integrin Subunit beta 5 (Itgb5) and A Disintegrin and Metalloproteinase Domain 8 (ADAM8) were more highly expressed in female than in male cells upon M-CSF treatment. With RANKL treatment, female cells expressed more integrin subunit alpha 4 (Itga4) and Itgb5 genes than male cells (Fig. 4). Second, the sex-related gene expression differences in adhesion between pre-osteoclast cells (M-CSF-treated) and OC differentiating cells (MRL-treated) were examined. In response to RANKL-treatment, the expression of Itga5 and Itgb5 decreased and the expression of Itga4 and Itgb3 increased in female cells compared to its control, while remaining unchanged in male cells (Fig. 4). In contrast, Itgav was increased in male cells, while remained unchanged in female cells, in response to RANKL-treatment when compared to their controls.

### 3.6 Osteoclast differentiation-associated genes

The sex-specific gene expression differences in differentiation-associated genes between pre-osteoclast cells (M-CSF treated) and OC differentiating cells (MRL-treated) cells were studied. The TNF Receptor Superfamily Member 11a (RANK), Spi-1 proto-oncogene (Spi1), interferon alpha and beta receptor subunit 1 (IFNAR1), transcription factor subunit (referred as cFOS or FOS), FOS proto-oncogene and activator protein 1 (AP-1) were expressed to a higher extent in female compared to male cells with M-CSF treatment, while TNF receptor superfamily member 11b (OPG) displayed a lower expression in females (Fig. 5). Along with Spi1, the expression of colony stimulating factor 1 receptor (Csf1R), phosphatidylinositol-4,5-bisphosphate 3-kinase catalytic subunit alpha (Pik3ca), and interferon gamma receptor 1 (IFNGR1) increased in females after RANKL treatment (Fig. 5).

Further, the sex-related gene expression differences in differentiation genes between pre-osteoclast (M-CSF treated) and OC differentiating (MRL-treated) cells were also examined. Upon RANKL treatment, there was an increase in the gene expression of Csf1R, Pik3ca and IFNGR1, while the expression of RANK and FOS was decreased in female compared to their control cells (Fig. 5). RANK expression increased upon RANKL treatment in male cells (Fig. 5). Of the functional genes, the expression of osteoclast stimulatory transmembrane protein (OCSTAMP), dendrocyte expressed seven transmembrane protein (DCSTAMP), nuclear factor of activated T-cells 1 (Nfatc1), acid phosphatase 5, tartrate resistant (ACP5) increased similarly in both sexes upon treatment. TNF receptor associated factor 6 (TRAF6), osteoclast associated Ig-like receptor (OSCAR), cathepsin K (Ctsk), calcitonin receptor (CalcR) and matrix metallopeptidase 9 (MMP9) increased in the female cells in response to RANKL treatment (Fig. 6). When performing a two-way ANOVA without the interaction effect, OSCAR, Ctsk, CalcR, and MMP9 were also upregulated in the male cells. The functional genes did not differ between the two sexes in the presence or absence of treatment (Fig. 6).

## 4 Discussion

Bone mass and architecture are sexually dimorphic in many species including mouse and man ((Galea et al., 2020) and referenced therein) and the gene expression in major bone compartments is also sexually dimorphic (Lu et al., 2019). These differences could be due to the varying bone resorption and formation activity, intertwined with role of the sex hormones, the availability of precursor cells, and the commitment of these cells to differentiate into active bone cells. In this study, we investigated sex-specific differences between adhesion, proliferation, and differentiation of bone marrow-derived macrophages into osteoclasts.

The female M-CSF-treated BMDM adhered faster than male cells in the first few hours after seeding and this early period is essential in determining the number of viable cells. M-CSF treatment supports the survival and proliferation of cells via the PI3K-AKT signaling pathway (Sullivan and Pixley, 2014; Tanaka et al., 1993), and promotes their differentiation into the macrophage-like lineage. Further, in females there was an increase in the expression of integrin and adhesion-associated genes, like Itga5 (associates with Itgb1) which binds fibronectin, Itgav (associates with Itgb3 and Itgb5) which binds vitronectin, osteopontin and bone sialoprotein (KEGG pathway: map04512), and ADAM8. These genes are essential for attachment and spreading of cells (Shima et al., 1995; Murrey et al., 2020; Ainola et al., 2009), which explains the higher adhesion of female compared to the male cells. Also, in treated female cells, the Itgavb5 form is expressed more than the Itgavb3 form. These two forms are structurally similar, and the substitution from the beta5 to beta3 subunit is important in switching from the bone macrophage-like to the osteoclast phenotype (Lane et al., 2005; Inoue et al., 2000), indicating that in this study the Itgavb5 form is more prominent in the female cells and makes them adhere more and differentiate less efficiently into osteoclasts than male cells. The regulation of these complexes in female cells supports our finding that female BMDM adhere better than male BMDM. It might also indicate that a larger proportion of the cells isolated from female mice have already been activated to macrophages (Chen et al., 2021), and therefore, they would be expressing more adhesion related genes and would be resistant to treatment inducing OC differentiation.

Once the cells have adhered, they need to survive and proliferate to be able to commit to their differentiation pathway depending on the stimulation. Females had a higher MRL/MCSF ratio while males differentiated more efficiently into osteoclasts upon RANKL stimulation. CSF1R binding to its ligand, M-CSF, is essential for the survival and proliferation of macrophages (Tanaka et al., 1993). CSF1R induces the RANK expression which is essential for osteoclast differentiation (Mun et al., 2020), and the PI3K-AKT signaling pathway is essential for their cell survival and proliferation (Sullivan and Pixley, 2014; Tanaka et al., 1993). The addition of RANKL inhibits proliferation and promotes differentiation of BMDMs into osteoclasts. In the current study, RANKL-treatment induced increase in proliferation ratio in the females on day 4 is related to an increase in the CSF1R and Pik3ca gene expression, and a decrease in the expression of the RANK gene in females. In contrast, in males, CSF1R and Pik3ca gene expression are not affected, while RANK expression is increased in response to RANKL-treatment. Taken together, on day 4, MCSF and PI3K signaling pathways promote the proliferative stage in female cells, while RANK-signaling promotes differentiation in male cells.

Higher expression of IFNAR1 and IFNGR1, while lower expression of FOS in females compared to the males after RANKL treatment could be indicative of negative regulation of RANK signaling and osteoclast differentiation. The binding of IFNγ to its receptor IFNGR1, inhibits the TRAF2/6 signaling, an essential complex downstream of RANK signaling in the osteoclast differentiation process (Lomaga et al., 1999; Takayanagi et al., 2000). Binding of IFNβ to its receptor IFNAR1negatively regulates osteoclast formation by inhibiting the RANKL-induced expression of cFOS (FOS), a transcription factor essential for promoting osteoclast differentiation (Takayanagi et al., 2002). cFOS-deficient mice are osteopetrotic due to lack of osteoclasts differentiation (Wagner, 2002). On the other hand, the pro-osteoclast differentiation gene, Spi1 (Tondravi et al., 1997), was higher in females upon RANKL treatment. The genes downstream of Spi1, essential in osteoclast differentiation and function, were upregulated to a similar extent in response to RANKL-treatment in both sexes. Interestingly, the expression of IFNAR1 and IFNGR1 were higher in female than male cells, indicating increased sensitivity for inhibition of osteoclast differentiation and more proinflammatory phenotype of the cells. Taken together, our results indicate that osteoclast differentiation is inhibited in the female, compared to male cells, through receptor signaling cascades at several levels, possibly due to reduced fusion of mononuclear osteoclast precursor cells.

In contrast, other studies have shown that female bone marrow cells differentiate faster into osteoclasts than male bone marrow cells (Mun et al., 2021; Paglia et al., 2016). Mechanistically, Mun and colleagues found that genes involved in immune and inflammatory pathways were differentially expressed in males and females in response to M-CSF and RANKL-treatment (Mun et al., 2021). These contrasting findings may in part be explained using different subsets of bone marrow cells. While we used all adherent cells, Mun and colleagues used the non-adherent fraction of the cultured bone marrow cells as well as the sorted CD45R^−^CD3^−^ CD11b^−/lo^ CD115^+^ osteoclast precursor cells. Moreover, macrophages activate quickly and the resulting cytokine signaling may make the cells resistant to other signals. Thus, one may speculate that different types of bone marrow cells facilitate different signaling pathways to promote differentiation into osteoclasts, and that activation of these pathways is sexually dimorphic. One could speculate that in a physiologically healthy state, the faster differentiation of male cells may not be relevant as the female cells most likely will catch up with the male cells eventually. However, in case of a pathological condition or fracture repair, this delayed osteoclast development might affect the repair process and remodeling in females (Haffner-Luntzer et al., 2021; Deng et al., 2020; Mehta et al., 2011).

A possible drawback of this study is the use of alpha MEM media with phenol red and FBS which was not charcoal stripped, providing a source of estrogen which might have partially inhibited the osteoclastogenesis by acting on estrogen receptors (ER). However, the serum was heat inactivated and the already low hormone levels in serum would be reduced further. The gene expression analysis data shows that the level of the ER-alpha receptor is higher in control male than female cells, thus if any estrogen mediated inhibition occurs, the male cells would probably have been affected more than female cells. Thus, our results may have been blunted by the presence of estrogen in the cell cultures.

## 5 Conclusion

In conclusion, adhesion and differentiation are sexually dimorphic in mouse bone marrow-derived macrophages. Gene expression analysis supports the notion that for these sexually dimorphic cellular effects, PI3K-Akt signaling pathway is important for adhesion and proliferation, while CSF1R and RANK signaling pathway are important for the differentiation. The regulation of these pathways by activators and inhibitors is also sexually dimorphic.

The following are the supplementary data related to this article.

Supplemental Table S1 Top 20 significantly enriched pathways in control male (CM) vs control female (CF) comparison and the genes involved in these pathways as per KEGG analysis. Pathways and genes important to our study are highlighted in red. Supplemental Table S1

Supplemental Table S2 Top 20 enriched pathways in treated male (TM) vs treated female (TF) comparison and the genes involved in this pathway as per KEGG analysis. Supplemental Table S2

Supplemental Table S3 Upregulated and downregulated genes in osteoclast differentiation as shown by the KEGG pathway. Red indicates upregulation and green indicates downregulation. Supplemental Table S3

Supplemental Fig. S1. (A) Proliferation of female and male bone marrow cells upon stimulation with M-CSF or a combination of M-CSF and RANKL measured on day 4. All data is adjusted to female (M-CSF) fluorescence output. (B) Osteoclast number of the technical replicates (per well) for each biological replicate. (S1A) Two-way ANOVA followed by Tukey's multiple comparison test was performed. Results are shown as median with interquartile range to show all points, *N* = 4–10.
Supplemental Fig. S2. Number of differentially expressed genes (DEG) in different comparison groups.
Supplemental Fig. S3. Enrichment of differentially expressed genes in KEGG pathways where -log10(p-adjusted value) > 1.3 is significant and represented with a dotted line. (A) control male (CM) vs control female (CF), (B) control female (CF) vs control male (CM), (C) treatment male (TM) vs control male (CM), and (D) treatment female (TF) vs control female.
Supplemental Fig. S4. Enrichment of differentially expressed genes in KEGG pathways for treatment females (TF) vs treatment males (TM) comparison. Image 1

## Funding

This work was supported by the 10.13039/501100002794Swedish Cancer Society #19 0165 Pj 01 H (GA), 10.13039/501100004359Swedish Research Council #2019-01295 (SW), 10.13039/501100004359Swedish Research Council #2017–01083 (TN), KI doctoral education grant #2018–1000 (TN).

## CRediT authorship contribution statement

**Suchita Desai:** Conceptualization, Data curation, Formal analysis, Investigation, Methodology, Validation, Visualization, Writing – original draft, Writing – review & editing. **Pernilla Lång:** Conceptualization, Formal analysis, Methodology, Supervision, Validation, Visualization, Writing – review & editing. **Tuomas Näreoja:** Formal analysis, Methodology, Supervision, Visualization, Writing – review & editing. **Sara H. Windahl:** Conceptualization, Formal analysis, Methodology, Supervision, Validation, Writing – original draft, Writing – review & editing. **Göran Andersson:** Conceptualization, Formal analysis, Funding acquisition, Methodology, Project administration, Supervision, Visualization, Writing – review & editing.

## Declaration of competing interest

None.

## Data Availability

Data will be made available on request.
