# Supplementary figures and images for: RANKL-dependent osteoclast differentiation and gene expression in bone marrow-derived cells from adult mice is sexually dimorphic
Source: Bone Rep. 2023 Jul 1;19:101697. doi: 10.1016/j.bonr.2023.101697 (PMC10359713; doi:10.1016/j.bonr.2023.101697)

**Figure S1**

**A**


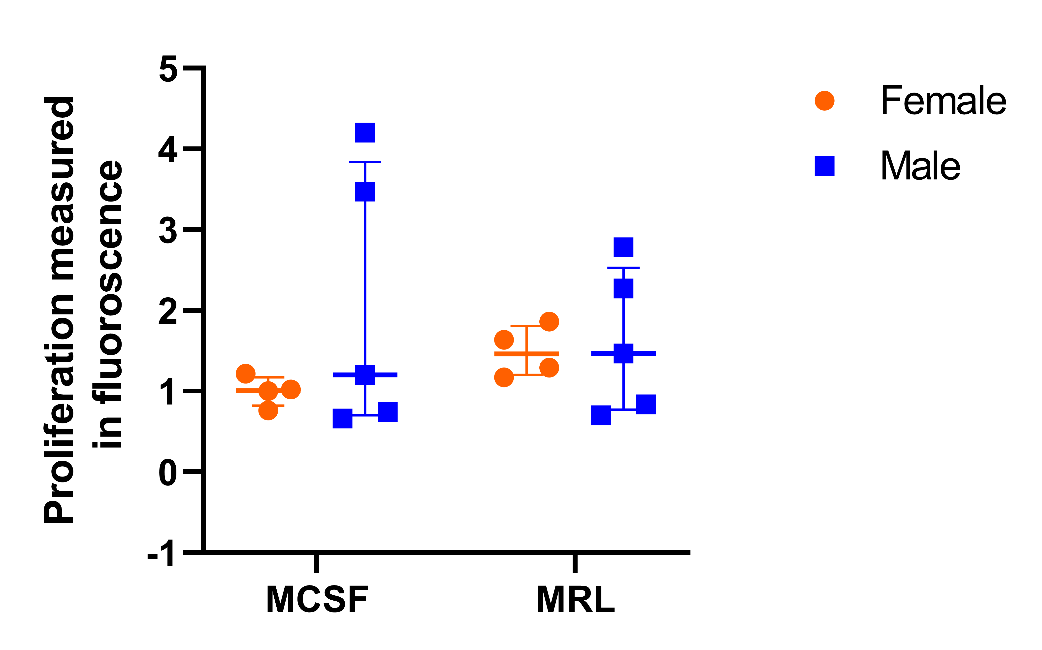


**B**

**Figure S2**

**
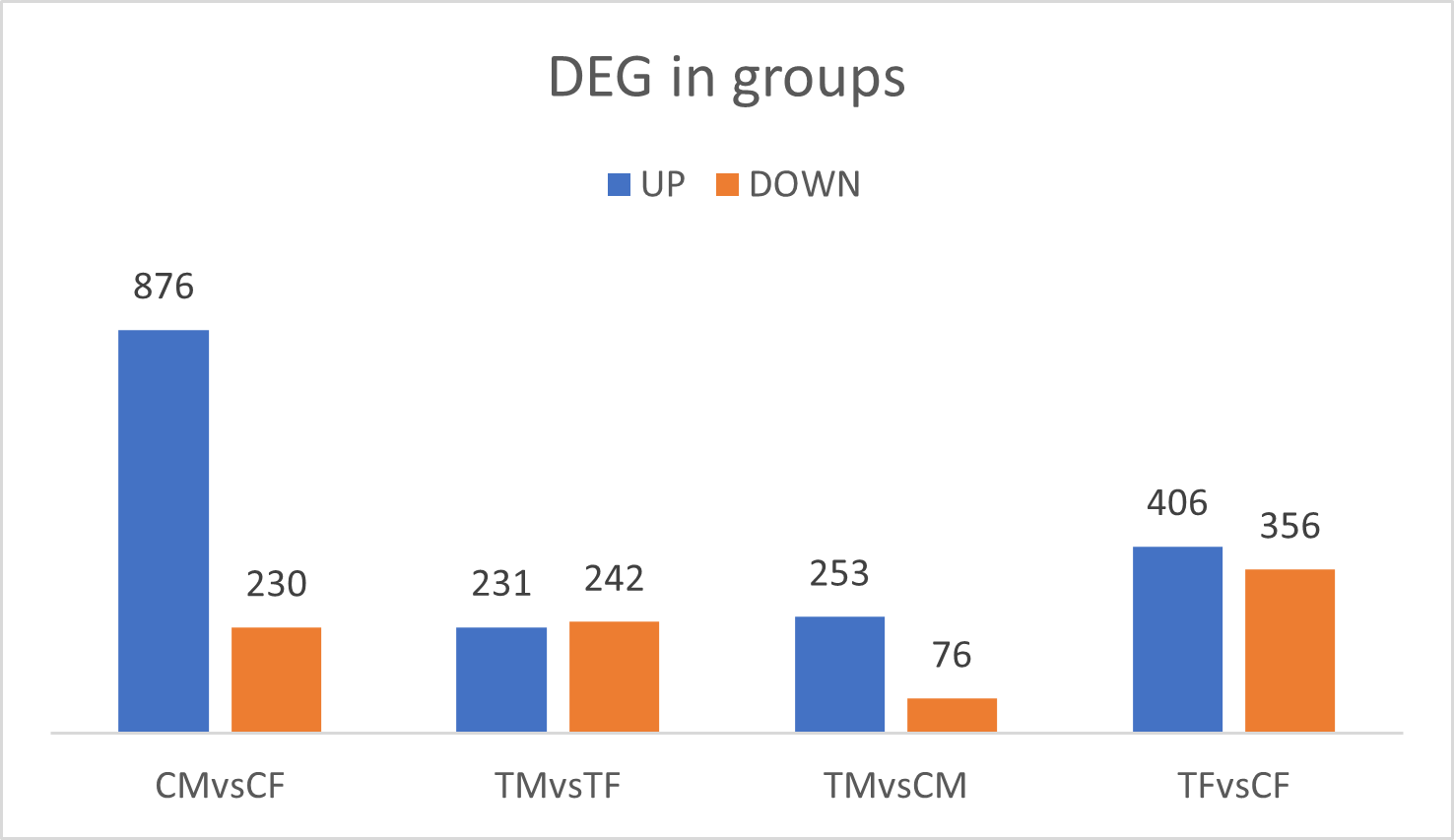
**

**Figure S3**

**B**

CF vs CM


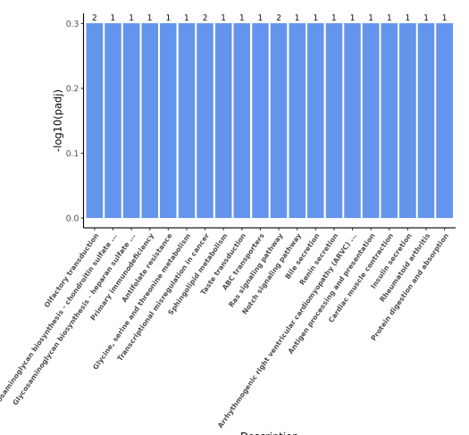


**A**

CM vs CF


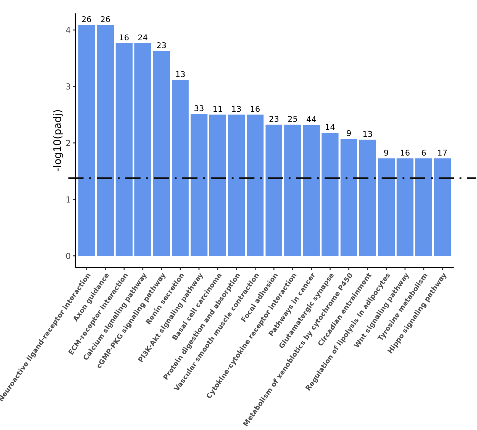


**C**

TM vs CM


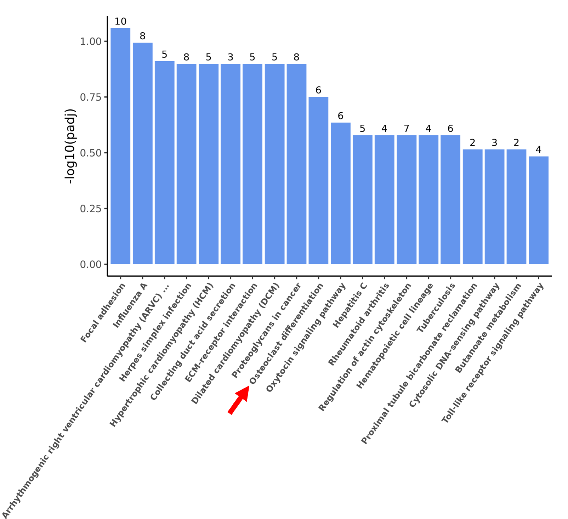


**D**

TF vs CF


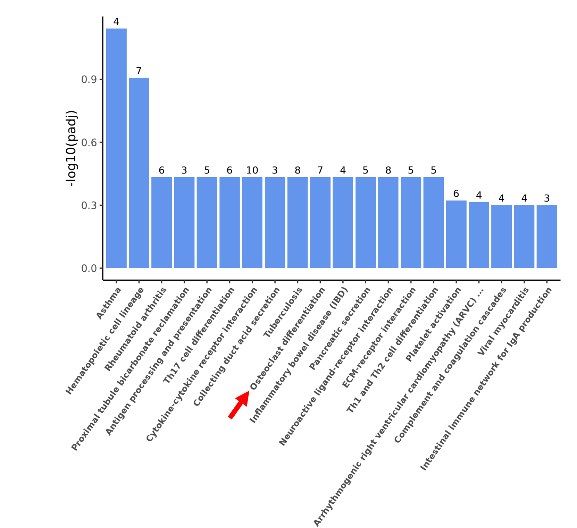


**Figure S4**

**
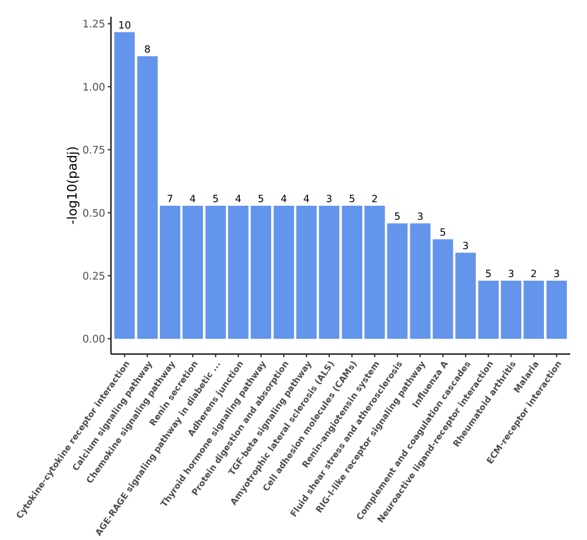
**

Supplement: Supplementary file 4 — Supplemental Fig. S1. (A) Proliferation of female and male bone marrow cells upon stimulation with M-CSF or a combination of M-CSF and RANKL measured on day 4. All data is adjusted to female (M-CSF) fluorescence output. (B) Osteoclast number of the technical replicates (per well) for each biological replicate. (S1A) Two-way ANOVA followed by Tukey's multiple comparison test was performed. Results are shown as median with interquartile range to show all points, N = 4–10. Supplemental Fig. S2. Number of differentially expressed genes (DEG) in different comparison groups. Supplemental Fig. S3. Enrichment of differentially expressed genes in KEGG pathways where -log10(p-adjusted value) > 1.3 is significant and represented with a dotted line. (A) control male (CM) vs control female (CF), (B) control female (CF) vs control male (CM), (C) treatment male (TM) vs control male (CM), and (D) treatment female (TF) vs control female. Supplemental Fig. S4. Enrichment of differentially expressed genes in KEGG pathways for treatment females (TF) vs treatment males (TM) comparison. [file mmc4.docx]
